# Structural and mechanistic insights into the herpes simplex virus type 1 helicase-primase primosome

**DOI:** 10.1038/s41421-025-00855-4

**Published:** 2025-12-10

**Authors:** Yaqi Wu, Ziyi Jiang, Xiaoling Chen, Danyang Li, Zhengyu Zhang, Changjiang Dong

**Affiliations:** 1 Department of Thyroid and Breast Surgery, Zhongnan Hospital of Wuhan University, School of Pharmaceutical Sciences, Wuhan University, Wuhan, Hubei China; 2 Key Laboratory of Combinatorial Biosynthesis and Drug Discovery, Ministry of Education, School of Pharmaceutical Sciences, Wuhan University, Wuhan, Hubei China; 3 The Cryo-EM Center, Core facility of Wuhan University, Wuhan University, Wuhan, Hubei China

**Keywords:** Replisome, DNA synthesis, Cryoelectron microscopy

## Abstract

DNA unwinding and primer synthesis are fundamental processes in genome replication. The human herpes simplex virus type 1 (HSV-1) helicase-primase forms a unique heterotrimeric primosome that is essential for viral DNA unwinding and primer synthesis and represents an ideal drug target. However, its molecular mechanism remains poorly understood. Here we report the cryo-electron microscopic structure of the primosome in complex with single-stranded DNA, ADP and Mg^2+^ to 3.47 Å resolution, which reveals that the primosome forms an unprecedented architecture in a fully open DNA binding groove between the helicase domains 1A and 2A–2B and that the primase subunit UL52 interacts extensively with the helicase subunit UL5 and accessory protein subunit UL8. Integrating mutagenesis, biochemical assays, structural analysis and 3D variability display analysis, we have identified the active sites of the ATPase, helicase and primase and critical interfaces between UL52, UL5 and UL8. Our work suggests that the primosome unwinds and translocates DNA via bidirectional rotation, and proposes a mechanistic model for DNA-dependent ATPase activation and alternating activity between helicase and primase. *Herpesviridae* family viruses pose significant threats to human health worldwide, and this trimeric assembly of primosomes is conserved. Our work provides a framework for understanding replication mechanisms across related viruses and for the rational design of broad-spectrum antivirals.

## Introduction

Human herpes simplex virus type 1 (HSV-1) is a highly versatile pathogen that infects > 67% of the world’s population of 0–49-year-olds and causes a wide range of diseases, from mild oral herpes to life-threatening encephalitis and disseminated infections^1–7^. Following primary infection, HSV-1 establishes lifelong latency in human trigeminal ganglia and can be periodically reactivated, causing recurrent disease; this reactivation is critical for transmission, though symptomatic recurrence is less common^8,9^. The clinical manifestations depend on the site of infection, the host’s immune status, and other factors^9,10^. HSV-1 infections represent a significant public health burden with underappreciated neurological consequences.

**Fig. 1:**
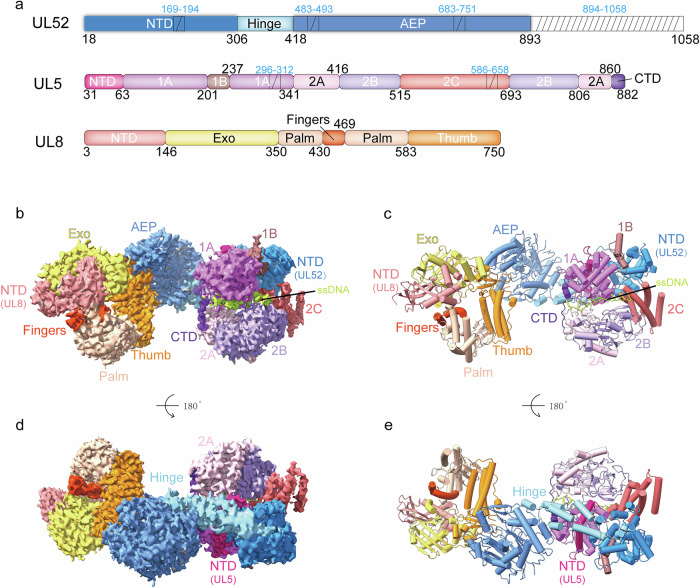
Overall structure of the HSV-1 primosome UL5–UL52–UL8 complex bound to ssDNA and ADP. **a** Domain organization of UL52, UL5, and UL8. Domains are colored distinctly. ssDNA is shown in lime. This color scheme is consistent throughout the study. The diagonal regions indicate areas poorly resolved in the density map. **b**, **d** Cryo-EM density maps of the primosome complex bound to ssDNA and ADP, with the domains outlined. **c**, **e** Cartoon representations of the primosome complex bound to ssDNA and ADP.

HSV-1 is an enveloped, double-stranded DNA (dsDNA) virus, which belongs to the *Alphaherpesvirinae* subfamily of the *Herpesviridae* family^11^. Its genome is ~152 kb and encodes over 80 proteins^12^. Replication of the HSV-1 genome, which occurs in the nucleus of infected cells, is an essential step in the viral life cycle. The virus encodes seven essential proteins that form the core replication machinery, each of which plays a distinct role in the replication process^12–14^. UL9 is an origin-binding protein that initiates the DNA replication, while UL29 (also called ICP8) is a single-stranded DNA (ssDNA)-binding protein that stabilizes unwound DNA^15^. The DNA polymerase UL30 and the processivity factor UL42 form the DNA replication machinery^16^, and UL5, UL8 and UL52 form a unique heterotrimeric helicase-primase complex (primosome)^17^, which is responsible for unwinding dsDNA and synthesizing short RNA primers^18,19^. UL5 is the helicase subunit (882 amino acids (aa)), UL52 is the primase subunit (1058 aa), and UL8 is an accessory protein (750 aa) that plays a regulatory role. This heterotrimer forms a primosome with a molecular weight of ~290 kDa^20,21^ (Fig. 1a). Recent structural studies have greatly advanced our understanding of the HSV-1 DNA polymerase and its proofreading function^22–24^. In contrast, structural insights into the helicase-primase complex remain limited.

Helicases are classified into six superfamilies (superfamily 1 (SF1)–SF6) on the basis of their conserved motifs and mechanism. The SF1 and SF2 are the two largest helicase families, and they normally exist in monomeric or dimeric forms, while SF3–SF6 are typically hexamers. The HSV-1 helicase UL5 subunit belongs to SF1 and is characterized by conserved ATPase motifs (Walker A/B) and 5′ → 3′ DNA unwinding activity^18^. The HSV-1 helicase-primase primosome differs from most well-studied hexameric DNA helicases by forming a unique heterotrimer^25^, yet the mechanisms governing its assembly and the coordination of its ATPase, unwinding, and primase activities remain largely unknown.

The DNA replication proteins of HSV-1 are important drug targets, and the current drugs acyclovir and foscarnet target the DNA replication machinery UL30–UL42 complex, but drug resistance against these drugs is becoming a serious problem, which requires the development of drugs based on the HSV-1 helicase-primase primosome^23,24,26^. The HSV-1 UL30–UL42 replication machinery is well characterized as a drug target. In contrast, the structural basis of the HSV-1 helicase-primase primosome has received less attention, thus limiting our understanding of how this three-component complex functions and how potential drugs might target it.

Here we report the 3.47 Å structure of the HSV-1 primosome, which captures its helicase domains (1A and 2A–2B) in a fully open conformation with ssDNA and ADP bound within the helicase. Combining mutagenesis, biochemical assays, structural analysis and 3D variability display analysis, we revealed the unique architecture of the HSV-1 helicase-primase and identified the subunit interfaces and the active sites of the ATPase, helicase and the primase, which are important for understanding the regulation and communication between the subunits. Our work provides a framework for novel drug development and understanding the helicase-primases of other *Herpesviridae* family viruses.

## Results

### The purified primosome exhibited DNA-dependent ATPase activity and helicase activity

The codon-optimized HSV-1 genes *ul5*, *ul8*, and *ul52* were synthesized and cloned into pFastBac vectors, with an 8× His-tag incorporated separately at the N-terminus of both UL5 and UL8 (Materials and Methods). The three component proteins were co-expressed in Bac-to-Bac expression system and purified using nickel affinity chromatography and size-exclusion chromatography (Supplementary Fig. S1a). Previous studies have reported that the primosome possesses DNA-dependent ATPase and helicase activities^25,27^. To assess the helicase activity of the purified primosome, we conducted assays similar to those used for the SF1B family helicase RecD^28^ (Materials and Methods). The results confirm that the purified protein possesses helicase activity (Supplementary Fig. S1b). To determine whether the purified primosome exhibited DNA-dependent ATPase activity, we conducted assays using a commercial kit (Materials and Methods). The results demonstrated that 250 nM purified primosome protein complex produced a ΔOD₆₂₀_nm_ = 0.579. Calculated per manufacturer’s protocol, this corresponds to an ATPase activity of 6.72 U/L (μmol/min/L), confirming functional ATP hydrolysis activity (Supplementary Fig. S1c, d). The protein’s ATPase activity was strongly stimulated after adding 10 μM ssDNA, with almost no activity detected in its absence. This result agrees with previous work showing that this protein requires DNA for ATPase activity (Supplementary Fig. S1d).

### The architecture of the primosome

The primosome was concentrated to 1.5 mg/mL and incubated with 2 mM ATP, 2 mM MgCl_2_ and 5 μM ssDNA for 2 h on ice. The sample was applied to a Quantifoil Au grid (Materials and Methods), and the cryogenic electron microscopy (cryo-EM) structure was determined to a resolution of 3.47 Å (HPF) (Supplementary Figs. S2a, b, S3a, b and Table S1). Due to inherent flexibility in the primase and helicase subunits, we performed local refinement with a focused mask to improve map quality, achieving a local resolution of 3.34 Å for these regions (HP) (Supplementary Figs. S2a, b, S3c, d and Table S1). The map showed clear densities for ssDNA and ADP (Supplementary Fig. S4a, e). The structure reveals that the stoichiometry of UL5, UL8, and UL52 is 1:1:1(Fig. 1b–e). The UL52 primase’s AEP domain connects to the UL8 accessory protein, while its N-terminal and middle hinge domains interact with the UL5 helicase. Notably, there are no direct contacts between UL8 and UL5 (Fig. 1b–e). The primosome measures 192.25 Å in length, 101.25 Å in height, and 83.89 Å in width. The model includes the UL5 helicase residues Gly31–Ala295, Cys313–Leu585, and Val659–Tyr882; the UL52 primase residues Val18–Asn168, Thr195–Ala482, Pro494–Pro682, Pro752–Ser893; and the UL8 accessory protein Thr3-Ala750 (Fig. 1a). The cryo-EM densities for the remaining primosome residues are invisible, suggesting that they might be flexible.

### The helicase structure

**Fig. 2:**
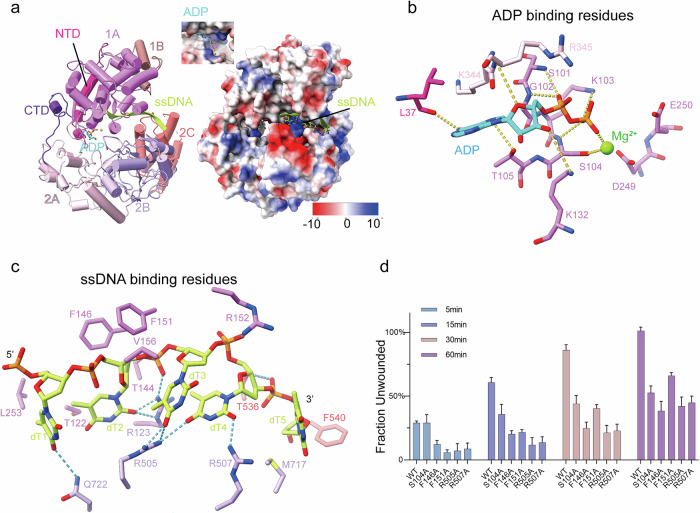
Structure of the HSV-1 helicase subunit UL5 bound to ssDNA and ADP. **a** Cartoon representation of UL5 colored by domains as the schematic. The ssDNA is shown in cartoon and the ADP and Mg^2+^ are shown in spheres; electrostatic surface potential of UL5 (blue: electropositive; red: electronegative). ssDNA and ADP are shown as spheres. **b** The ATPase active site of UL5. ADP is cyan; Mg^2+^ is green. Polar contacts are indicated by yellow dashed lines. **c** Key residues binding the ssDNA. Polar contacts are indicated in blue dashed lines. **d** Helicase activity of the HSV-1 UL5–UL52–UL8 complex. UL5 mutations (Ser104Ala, Phe146Ala, Phe151 Ala, Arg505Ala, Arg507Ala) significantly reduced the complex’s helicase activity in vitro compared to wild type (WT). The data represent mean ± SD of three independent experiments.

The UL5 helicase consists of seven domains in total: the N-terminal domain (NTD), domains 1A, 1B, 2A, 2B, 2C, and the C-terminal domain (CTD) (Fig. 1a; Supplementary Fig. S6a). The NTD contains a loop and a helix. Domain 1A forms a β-sandwich structure with five parallel β-strands in the middle, flanked by two α-helices at the top and seven α-helices at the bottom, exhibiting a RecA-like fold. Domain 1B is composed of two α-helices arranged in a wedge shape. Domain 2A forms another β-sandwich with four parallel β-strands at its core, two α-helices at the top, and four α-helices at the bottom, also displaying a RecA-like fold. Domain 2B consists of two β-sheets surrounded by five α-helices. The first β-sheet contains five antiparallel β-strands wrapped around three α-helices, while the second β-sheet comprises 10 β-strands folded into a barrel-like structure resting on two α-helices, exhibiting an SH3-like fold. Domain 2C forms an α-helix bundle with four helices adjacent to domain 2B. The CTD contains two loops and one α-helix (Fig. 2a; Supplementary Fig. S5).

UL5 belongs to the SF1B subfamily, which includes well-characterized helicases such as the T4 phage Dda^29–33^ (Supplementary Fig. S6b) and human Pif1^34–39^ (Supplementary Fig. S6d). Structural alignment of the T4 phage Dda (PDB: 3UPU)^31^ and human Pif1 (PDB: 6HPQ)^34^ with UL5 (Supplementary Fig. S6c–f) revealed distinct structural differences, with root-mean-square deviations (RMSD) of 3.989 Å and 4.181 Å over 265 and 235 aligned residues, respectively. Compared to these two related proteins, UL5 is notably larger, with a surface area of 35,723 Å². In contrast, Pif1 and Dda have surface areas of only 16,733 Å² and 19,863 Å², respectively. To better visualize the structural differences between UL5 and other SF1B helicases, we performed a separate structural alignment of its domains 1A, 2A, and 2B with their corresponding domains in Pif1. Structural alignment reveals a conserved RecA-like fold in the 1A domains (RMSD = 1.103 Å over 57 pruned atom pairs), although UL5 contains several additional structural elements distal to the ATPase active site (Supplementary Fig. S6g). The 2A domains show even higher structural similarity, with an RMSD of 0.800 Å across 12 matched atoms (Supplementary Fig. S6h). In sharp contrast, the 2B domains are structurally divergent and could not be reliably aligned using automated methods (Supplementary Fig. S6i, j). In addition to differences in their molecular weights, the significant structural variations between these domains likely arise from the distinct functional states of Pif1 and UL5, as they are bound to different substrates.

### The ATPase active site

The ATPase active site of UL5 helicase contains densities corresponding to ADP and magnesium ion, suggesting that the ATP has been cleaved to ADP (Supplementary Fig. S4e). Adenine is stabilized by Leu37, Lys344 and Thr105, and ribose interacts with Arg345 and Lys132, while the α-phosphate and β-phosphate are coordinated by residues Ser101, Gly102, Lys103 and Ser104. The magnesium ion is chelated by residues Ser104 and β-phosphate, while the catalytic residues Asp249 and Glu250 are located around the β-phosphate (Fig. 2b; Supplementary Fig. S4f). Structural superposition of the human helicase Pif1 (PDB: 6HPQ)^34^ to the UL5 helicase revealed that the AMPPNP and the ATP binding/catalytic residues Ser235, Asp306, Glu307 and Arg381 of the human helicase Pif1 occupy positions analogous to those of ADP, Ser104, Asp249, Glu250 and Arg345 of UL5 in complex with the ADP (Supplementary Fig. S6d–f), suggesting that the ATP binding/catalytic residues are evolutionarily conserved. Single mutants Gly102Val and Lys103Ala significantly reduced the ATPase activity, while Asp249Ala and Glu250Ala abolished the ATPase activity^40^. A transient replication complementation assay revealed that the mutants Gly102Val, Lys103Ala failed to synthesize the viral DNA^41^. These data corroborate the structural insights. We generated the UL5 Ser104Ala mutant, co-expressed it with UL52 and UL8, and tested the helicase activity of the mutant complex. This resulted in a significantly reduced percentage of DNA unwinding, confirmed that Ser104 is a critical residue for ATPase and helicase function (Fig. 2d).

### ssDNA binds within the fully open interdomain groove of the helicase

**Fig. 3:**
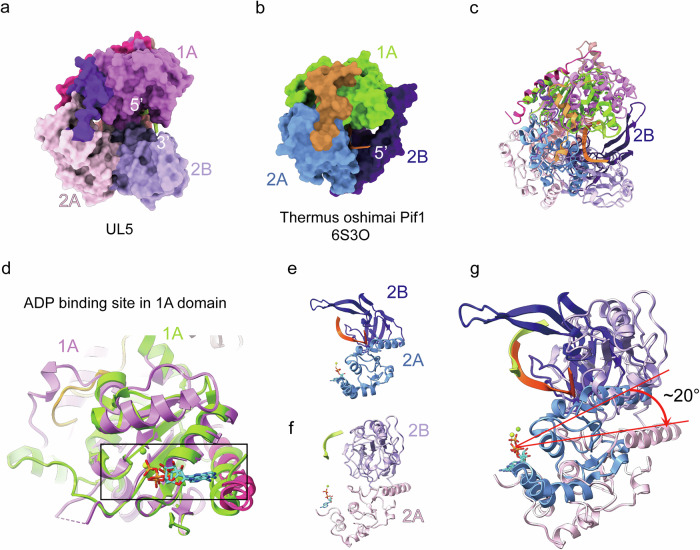
The helicase subunit UL5 adopts a fully open conformation. **a** Cartoon representation of UL5 colored by domain (schematic). Domains 1A, 2A, and 2B are explicitly highlighted. **b** Cartoon representation of *Thermus oshimai* Pif1 (ssDNA/ADP-bound; PDB: 6S3O). Domains 1A, 2A, and 2B are explicitly highlighted. **c** Superposition of Pif1 onto UL5 highlights divergent conformations in the 2A and 2B domains. **d** Close structural alignment of the 1A domains and ADP-binding sites between UL5 and Pif1. **e** Conformation of the Pif1 2A–2B domains. **f** Conformation of the UL5 2A–2B domains. **g** Domain 2A in UL5 exhibits an ~20° rotation relative to Pif1. UL5 domain 2B lacks the 1A-proximal loop observed in Pif1.

In the UL5 helicase structure, a highly positively charged groove between the 1A and 2A–2B domains accommodates the density corresponding to ssDNA as PolyT does (Fig. 2a; Supplementary Fig. S4a), which was used in sample preparation (Materials and Methods). Key residues, including Phe146, Phe151, Val156, Arg123, Arg152, Arg505, Arg507, Phe540, Thr536, Met717, Gln722, Thr144, Thr122 and L253 interact with the ssDNA (Fig. 2c; Supplementary Fig. S4b–d). Hydrophobic contacts involve residues Val156, Phe146, Phe151, Leu253, Thr122, Phe540, and Met717, which engage the deoxyribose and nucleobases. Hydrogen bonds are formed by Gln722, Arg123, Thr144, Arg505, Arg507, and Thr536 with the nucleobases and phosphate groups. Key salt bridges are mediated by Arg123 and Arg152 to the phosphate groups (Supplementary Fig. S4d). To validate whether the DNA binding residues are important for the helicase activity, we generated UL5 mutants (Phe146Ala, Phe151Ala, Arg505Ala, Arg507Ala), co-expressed each with UL52 and UL8, and purified the resulting complexes. Helicase activity assays were then performed on these complexes, alongside the ATPase mutant Ser104Ala. These assays showed that all mutants exhibited significantly reduced helicase activity (Fig. 2d), confirming the critical role of these DNA-binding residues. Superimposing the structure of *Thermus oshimai* Pif1 complexed with ssDNA and ADP (PDB: 6S3O)^42^ onto the UL5 helicase structure revealed a distinct DNA-binding state and a fully open conformation, in which domains 1A and 2A–2B create an extended groove that positions the ssDNA (Fig. 3a–c). Compared with Pif1, UL5’s 2A domain rotates ~20° relative to 1A (centered on the ADP-binding site), while the 2B domain adopts a distinct conformation, repositioning away from 1A and lacking the 1A-proximal loop. This rearrangement creates a widened interdomain groove between domains 1A and 2A–2B. (Fig. 3d–g). Consistent with these structural rearrangements, clear ssDNA density is resolved in the 1A–2B groove but becomes disordered near the displaced 2A domain. This unreported conformational state in SF1B helicases provides mechanistic insights into DNA binding dynamics.

### Primase structure

The primase UL52 subunit comprises four distinct domains: the NTD, hinge domain, archaea-eukaryotic primase (AEP) domain, and zinc-binding domain (Fig. 1a; Supplementary Fig. S8a). The NTD features a core β-sheet formed by four antiparallel β-strands, flanked by five α-helices on one side and six α-helices on the other, connected by a loop to form a characteristic α/β fold. The hinge domain consists of three elongated α-helices that assemble into a helical bundle structure. The AEP domain contains three α/β motifs: the first β-sheet comprises three antiparallel β-strands; the second contains four antiparallel β-strands; the third consists of five antiparallel β-strands. These β-sheets are surrounded by a total of 13 α-helices (Supplementary Fig. S7). Dali server^43^ analysis revealed structural similarity between the UL52 AEP domain and both the human primpol (PDB: 5L2X)^44^ and mpox primase domain (PDB: 8WGY)^45^, with RMSD values of 2.715 Å and 3.002 Å over 205 aligned residues and 169 aligned residues, respectively (Supplementary Fig. S8b–d). Notably, the cryo-EM density for the zinc-binding domain was not resolved, indicating structural flexibility in our reconstruction.

### The primase active site

The primase UL52 synthesizes RNA primers for DNA synthesis. This process requires the primase to bind pyrimidine-rich DNA templates and nucleotide triphosphates (NTPs) to generate short RNA primers (2–4 nucleotides (nt))^27,46^. However, no densities corresponding to ssDNA or NTPs were observed in the UL52 structure. To identify the UL52 primase active site, we superimposed the structure of human PrimPol in complex with dsDNA, dATP, and calcium (PDB: 7JKP)^47^ onto the UL52 AEP structure. This analysis revealed that the human Primpol nucleotide binding residues Lys297, Arg291, and Arg76 and the catalytic residues Glu116, Asp114 and Asp280 are superimposed to residues Lys829, Arg823, Arg537, Asp630, Asp628 and Asp812 of UL52 (Supplementary Fig. S8e–g). The dATP in the PrimPol structure, mimicking an incoming NTP, occupies a pocket formed by UL52 residues Lys829, Arg823, Arg537, Asp630, Asp628, and Asp812, suggesting that Lys829, Arg823, and Arg537 likely mediate NTP binding, and that Asp630, Asp628, and Asp812 may constitute the catalytic triad for primer synthesis. The UL52 mutant Asp628Gln completely abolished the primase activity^48^, while the Asp626Ala and Asp630Ala mutants dramatically reduced the primase activity^49^. These reports are consistent with our structural observations and comparative analysis findings.

### Structure of the accessory protein

The accessory protein subunit UL8 comprises five domains: an NTD, an exonuclease domain, a palm domain, a finger domain and a thumb domain (Fig. 1a; Supplementary Fig. S10a). These domains interact with each forming a circular structure with a central hole. The NTD contains 2 β sheets (each with 3 antiparallel β-strands) and 3 α-helices. The exonuclease domain features 2 β-sheets, one with 2 antiparallel β-strands and the other with 8 antiparallel β-strands, surrounded by 7 α-helices. The palm domain contains 2 back-to-back β-sheets, each with 4 antiparallel β-strands, and 5 α-helices at the opposite end. The finger domain has 2 α-helices. The thumb domain consists of 2 motifs, motif 1 having 3 anti-parallel β-strands and 3 α-helices, and motif 2 consisting of 4 α-helices (Supplementary Fig. S9).

The overall architecture of UL8 resembles that of the *Enterobacteri* phage RB69 GP43 DNA polymerase (PDB: 1Q9X)^50^ (Supplementary Fig. S10b), *Thermococcus gorgonarius* DNA polymerase (PDB: 2XHB)^51^ (Supplementary Fig. S10c) with an RMSD of 4.7 and 4.92 over aligned 490, and 325 residues, respectively. Although homology-based analysis predicted partial similarity between UL8 and B-family polymerases, UL8 exhibits neither DNA polymerase nor exonuclease activity^35,52^. Comparative analysis of the surface charge distribution revealed significant divergence between UL8 and B-family polymerases (Supplementary Fig. S10d–f). Both RB69 GP43 DNA and *T. gorgonarius* DNA polymerases exhibit a more pronounced clustering of positive charges for DNA binding. This structural feature suggests that UL8 cannot bind template-primer forked DNA such as canonical B-family polymerases, consistent with prior findings that UL8 specifically binds ssDNA exceeding 50 nt in length^53,54^.

### Extensive interactions between helicase UL5 and primase UL52

**Fig. 4:**
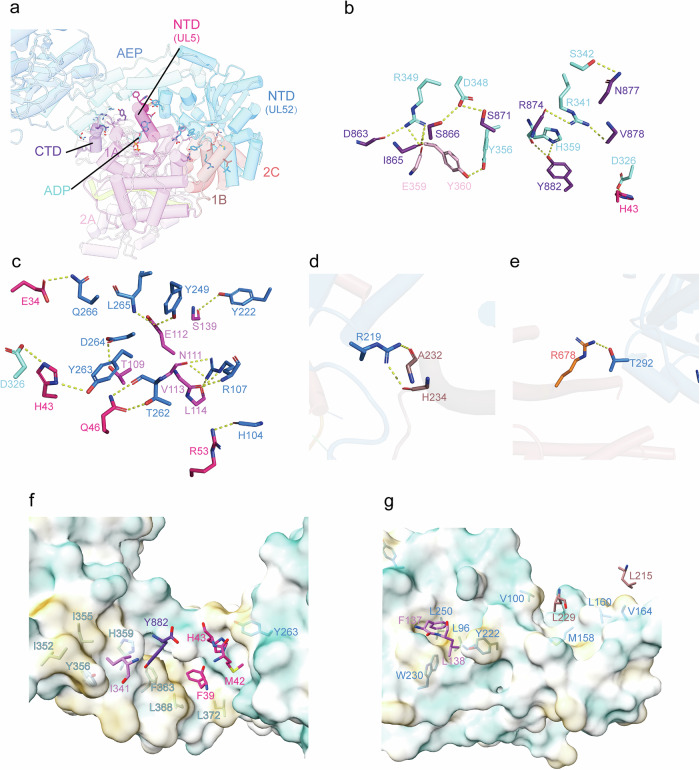
Interaction between the helicase subunit UL5 and primase subunit UL52. **a** NTD and hinge domain of UL52 envelop the helicase UL5, forming an extensive interaction interface. **b** Polar contacts between the UL52 hinge domain and UL5. The yellow dashed lines indicate hydrogen bonds/salt bridges. **c** Polar contacts between the UL52 NTD domain and UL5. The yellow dashed lines indicate hydrogen bonds/salt bridges. **d** Polar contact between the UL52 NTD domain and the UL5 CTD domain. **e** Polar contact between the UL52 NTD domain and the UL5 2C domain. **f**, **g** Hydrophobic interactions at the UL5–UL52 interface. UL52 is surface-rendered (ChimeraX, Coulombic Surface Coloring). Key interacting residues shown as sticks.

The NTD and the hinge domain of the primase UL52 envelop the helicase NTD, 1A, 1B, 2C and the CTD domains of the helicase UL5, forming an extensive interaction interface (Fig. 4a). The interface of the two proteins is 2801.1 Å^2^. In particular, primase UL52 residues Arg341, Arg349, Asp348, Asp326 (Fig. 4b), Arg107, Tyr263, Tyr249 (Fig. 4c), Arg219 (Fig. 4d), Thr292 (Fig. 4e) and hydrophobic residues Ile352, Ile355, Tyr356, His359, Phe363, Leu368, Tyr263 (Fig. 4f), Trp230, Tyr222, Leu96, Leu250, Met158 Val100, Leu160 and Val164 (Fig. 4g), interact with the residues Arg874, Asp863, Ser871, His43 (Fig. 4b), Asn111, His43, Glu112 (Fig. 4c), His234 (Fig. 4d) and Arg678 (Fig. 4e) of helicase UL5 and hydrophobic residues Ile341, Tyr882, Phe39, His43, Met42 (Fig. 4f), Phe137, Leu138, Leu229, Leu215 (Fig. 4g). The helicase ATP binding site is close to the interaction interface of the helicase and the primase subunits (Fig. 4a). Pervious studies have shown that the UL52 NTD residues Arg367–Arg420 interact with UL5, while the middle domain of UL52 residues Gly422–Leu887 interact with the residues from the UL8 CTD^19^, consistent with our structural observations.

### Interaction of the primase UL52 and the accessory protein UL8

**Fig. 5:**
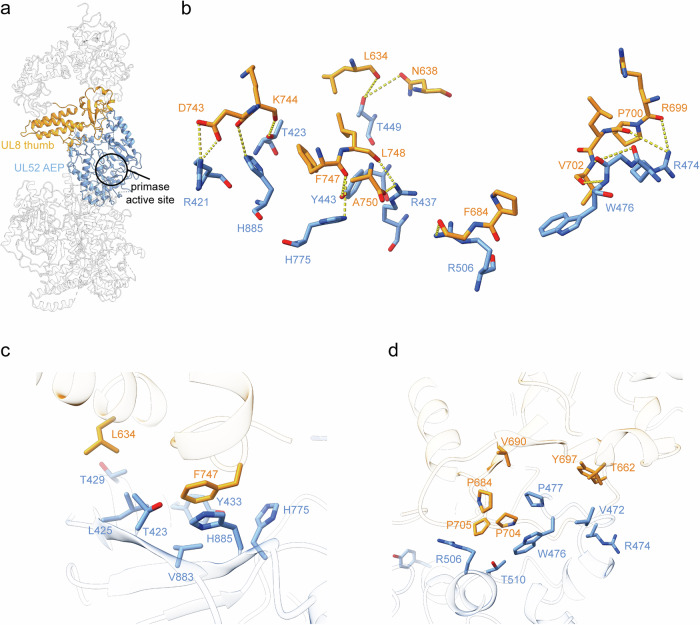
Interaction between the UL8 and UL52. **a** UL8 thumb domain interacts with the UL52 AEP domain. Both domains are shown in cartoon representation using schematic coloring. **b** Polar contacts (hydrogen bonds/salt bridges) between the UL52 AEP and UL8 Thumb domains, as indicated by yellow dashed lines. **c**, **d** Hydrophobic interactions at the UL8–UL52 interface.

The primase UL52 and the accessory protein UL8 interact through the primase AEP domain and the accessory protein thumb domain, forming an interface, with a total buried surface area of 1203.3 Å² (Fig. 5a). In particular, the primase residues Arg421, His885, Tyr443, His775, Arg437, Arg506, Trp476, and Arg474 form polar contacts with the accessory protein UL8 residues Asp743, Lys744, Phe747, Leu748, Phe684, Val702, Pro700, and Arg699 (Fig. 5b). The hydrophobic residues Thr429, Leu425, Thr423, Val883, His885, Tyr433, His775 (Fig. 5c), Arg506, Thr510, Trp476, Pro477, Val472, and Arg474 (Fig. 5d) of UL52 interact with L634, Phe747 (Fig. 5c), Pro705, Pro704, Pro684, Val690, Tyr697, and Thr662 (Fig. 5d) of UL8. The primase active site is positioned adjacent to the interaction region between the primase and the accessory protein UL8 (Fig. 5a).

### Dynamics of the primosome

To investigate the dynamics of the primosome, we analyzed the primosome structure through 3D variability display in CryoSPARC^55^, which revealed different conformational states (Supplementary Video S1). The UL5 1A and 2A–2B domains rotate in the clockwise direction to shrink and narrow the DNA-binding groove, while the DNA-binding groove becomes widened and enlarged when these two domains rotate in the counterclockwise direction. The primase NTD domain moves in the same direction as that of the helicase. In contrast, the accessory protein subunit slightly moves in the opposite direction of that of the helicase. The helicase subunits along with the primase NTD and hinge domain exhibit significantly greater mobility than the primase AEP domain and accessory subunits. This structural flexibility within the helicase complex is functionally consistent with UL5’s mechanism of harnessing ATP hydrolysis to power DNA unwinding. Notably, conformational changes in the primase active site accompany helicase subunit rotation, suggesting that primase and helicase activities might be functionally coordinated. The primosome accessory protein can bind DNA longer than 90 nt and stimulate both helicase and primase activities^53,54^. Deficiency of the helicase activity stimulates the primase activity^40^, suggesting that these two activities are negatively regulated, potentially through the conformational changes that we observed. We hypothesize that the helicase conformational changes could allosterically regulate the primase activity.

## Discussion

The HSV-1 trimeric helicase-primase primosome is a critical component of the HSV-1 DNA replication machinery and plays essential roles in both dsDNA unwinding and primer synthesis. Unlike other SF1 family members, which typically exist as monomers or dimers, the HSV-1 primosome adopts a unique heterotrimeric form, representing a novel structural configuration within this protein family^18^. We determined the cryo-EM structure of the HSV-1 trimeric primosome at near-atomic resolution, which revealed that the primase UL52 subunit utilizes its N-terminal and hinge domains to interact with the helicase NTD, 1A, 2A, 2B, 2C and CTD domains, while the primase AEP domain connects with the thumb domain of the accessory protein UL8. This structural arrangement differs significantly from reported SF1 helicase conformations and those of other helicase superfamilies. The structure captures the primosome in a post-catalytic state, with ADP and magnesium ions bound at the ATPase site in the presence of ssDNA. This structural information enables the identification of key residues for ATP binding and DNA binding, as well as the interfaces among its three subunits. Through further structural comparisons, we have identified the catalytic residues of the ATPase and the primase active site. These findings provide a structural foundation for a deeper understanding of the HSV-1 replication machinery and offer further insights into the functional mechanisms of DNA helicases within the SF1B family.

### Connection of the ATPase active site with ssDNA binding and drug resistance

**Fig. 6:**
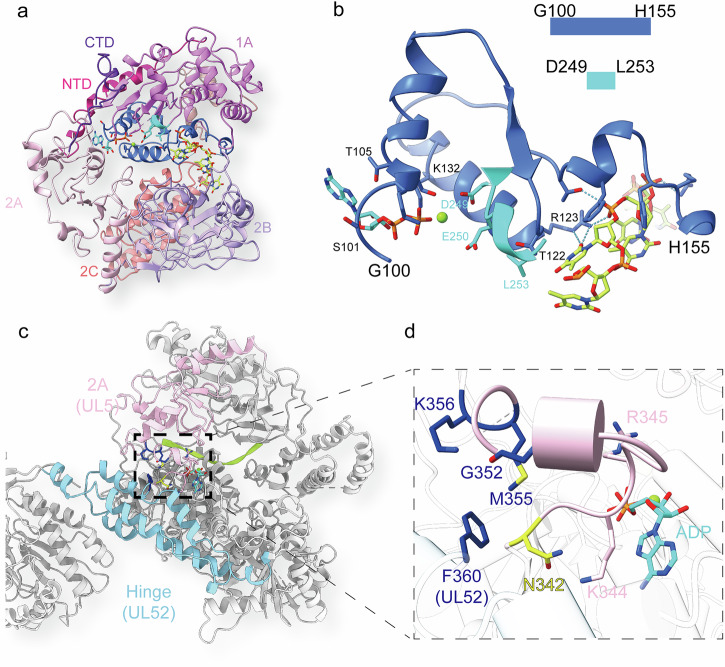
Structural basis linking UL5 ATPase to ssDNA binding and to drug resistance. **a** Functional coupling loops connecting ADP-binding (cyan) and ssDNA-binding (deep blue) sites highlight mechanistic linkages. **b** DNA-binding residue Leu253 is located in the same cyan loop as the catalytic residues Asp249 and Glu250. The DNA-binding residue Arg123 resides in the same deep blue loop as the ATP-binding residues Ser101–Thr105. **c**, **d** Common resistance mutation sites for pritelivir and amenamevir are located near the ADP-binding site. Dark blue/yellow sticks show resistance mutations; pink sticks show ADP-binding residues.

The HSV-1 primosome exhibits DNA-dependent ATPase activity, similar to the well-characterized *T. oshimai* Pif1 helicase. Structural analysis of *T. oshimai* Pif1 in three states: apo form (PDB: 6S3E), polyT-bound state (PDB: 6S3P), and polyT/ADP-bound state (PDB: 6S3O), demonstrates that polyT binding repositions the catalytic residues Asp171 and Glu172 into their active conformations. Concurrently, the ATP-binding loop (containing Thr95, Gly96, and Lys97) transitions from a closed to semi-open state, with ATP binding ultimately inducing the full opening of this loop to facilitate nucleotide binding^42^. Our structural studies of primosome revealed that the DNA-binding residue Leu253 is located in the same loop as the catalytic residues Asp249 and Glu250, and that the DNA-binding residue Arg123 is located in the same loop as the ATP-binding residues Ser101, Lys103, Ser104, and Thr105 (Fig. 6a, b). These observations suggest that HSV-1 primosome likely employs a DNA-dependent ATPase mechanism similar to that of *T. oshimai* Pif1, where DNA binding induces conformational changes in both the catalytic and ATP-binding loops, and ATP hydrolysis and subsequent release drive structural rearrangements in DNA-binding residues. These changes promote rotation of the 1A and 2B domains of the UL5 helicase, coupling ATP hydrolysis to 5′-3′ DNA unwinding and translocation. This mechanistic conservation between *T. oshimai* Pif1 and HSV-1 helicase-primase highlights the evolutionary preservation of DNA-dependent ATPase activation in these helicases.

The current research on HSV-1 primosome-targeting therapeutic drugs primarily focuses on pritelivir^56^ and amenamevir^57^. To investigate whether our structural data can shed light on the mechanism of action and resistance mutations of these two drugs, we identified reported resistance mutation sites including pritelivir (Asn342Lys^58^, Gly352Val, Met355Thr, and Lys356Gln^59^ in UL5) and amenamevir (Gly352Cys, Met355Thr, and Lys356Gln in UL5, as well as Phe360Val/Cys in UL52^60^). Our structural localization reveals that these resistance sites in UL5 are all situated within the 2A domain, while the Phe360 in UL52 are also located nearby, strongly suggesting that this region may constitute the drug-binding pocket (Fig. 6c). Moreover, these drug resistance mutation sites are all located near the ADP-binding site, with residues including Asn342, Gly352, Met355 and Lys356 residing on the same loop as Lys344 and Arg345, which interact directly with ADP (Fig. 6d). We therefore hypothesize that both pritelivir and amenamevir exert their antiviral effects by binding to the protein, altering the conformation of the ATPase active site, and thereby affecting both ATPase and helicase activities.

### DNA unwinding and primase activity

The dsDNA needs to unwind into the ssDNA for DNA replication. Our structure captures the primosome with its helicase DNA-binding groove in a fully open conformation. Compared with the *T. oshimai* Pif1 complexed with ssDNA and ADP (PDB: 6S3O), the groove between domains 1A and 2A–2B undergoes enlargement and contraction through rotation of 2A and conformational changes in 2B, which might contribute to the flexibility of the zinc-binding domain of primase UL52 in our structure. This dynamic behavior is consistent with the 3D conformational flexibility captured in Supplementary Video S1.

Dynamic analysis revealed that the helicase rotates in both counterclockwise and clockwise directions to unwind DNA between the helicase 1A and 2A–2B groove, while the primase subunit moves in the same directions as that of the helicase subunit (Supplementary Video S1). The loss of ATPase activities stimulated primase activities, indicating that these two active sites may operate through an alternating mechanism for helicase and primase activities, similar to the reported alternative mechanism for primase and DNA polymerase^17^.

HSV-1 belongs to the *Herpesviridae* family, which comprises more than 100 viruses classified into three subfamilies: *Alphaherpesvirinae*, *Betaherpesvirinae*, and *Gammaherpesvirinae*^18^. The heterotrimeric primosome arrangement is conserved across these superfamilies^18^. Our work provides an important platform for studying the primosome mechanism and function of this virus family, which is crucial for novel drug discovery targeting herpsviridae viruses and other pathogenic DNA viruses.

## Materials and methods

### Cloning and expression of the UL5–UL52–UL8 complex and its mutants

The full-length gene fragments encoding HSV-1 UL5, UL52, and UL8 were synthesized and codon-optimized for the Bac-to-Bac expression system. The optimized UL5 gene sequence was fused with an N-terminal His8 tag, followed by a TEV protease cleavage site, and inserted into the pFasBac1 transfer vector. The UL8 gene sequence was similarly fused with an N-terminal His8 tag, followed by a TEV protease cleavage site, and inserted downstream of the polyhedrin promoter in the pFastBac Dual vector, while the UL52 gene sequence was placed downstream of the p10 promoter. Positive baculovirus recombinants were identified and isolated after transformation into DH10Bac component cells (Solarbio) and growth on selective LB agar plates. The recombinant baculovirus DNA was then purified and used to transfect *Spodoptera frugiperda* 9 (Sf9) cells to generate a baculovirus stock, which could be subsequently amplified and used for protein expression in insect cells. The two virus stocks, each amplified separately, were mixed in a 1:1 ratio and used to co-infect Hi5 insect cells at a density of 2 × 10⁶ cells/mL for the co-expression of UL5–UL52–UL8 proteins at 27 °C for 50 h. UL5 mutations (Phe146Ala, Phe151Ala, Arg505Ala, Arg507Ala, and Ser104Ala) were generated via site-directed mutagenesis. Virus stocks containing these UL5 mutants were prepared as previously described and then mixed with UL52–UL8 virus stocks to produce mutant complexes.

### Purification of the UL5–UL52–UL8 complex protein

Unless otherwise specified, all procedures were carried out at 4 °C. Hi5 cells expressing the UL5–UL52–UL8 complex were harvested, washed, and resuspended in lysis buffer (25 mM HEPES, pH 7.5, 300 mM NaCl, 10% glycerol, and 1 mM TCEP) supplemented with an EDTA-free protease inhibitor cocktail, and then lysed by sonication. The lysate was clarified by centrifugation at 20,000× *g* for 30 min at 4 °C and filtered through a 0.45-μm filter before being loaded onto Ni-NTA resin. The resin was washed with Buffer A (20 mM HEPES, pH 7.5, 300 mM NaCl, 10% glycerol, and 40 mM imidazole), and the bound protein was eluted with Buffer A containing 300 mM imidazole. For further purification, the eluate was concentrated and applied to a size-exclusion chromatography column (Superose 6 Increase 10/300 GL, Cytiva) equilibrated with Gel Filtration Buffer H (20 mM HEPES, pH 7.5, 150 mM NaCl, and 5% glycerol), using an AKTApure protein purification system. Proteins were analyzed by SDS-PAGE, concentrated using a centrifugal concentrator, and stored at –80 °C until further use. The purification and analysis procedures for the mutant complexes were identical to those used for the WT protein (Supplementary Fig. S11a–e). All proteins were concentrated to 1.5 mg/mL, aliquoted, and stored at –80 °C until use.

### ATPase assays

The ATPase activity of the UL5–UL52–UL8 primosome was measured by quantifying the rate of ATP hydrolysis using the malachite green assay (BioAssay Systems, DAT-G-200), following the manufacturer’s instructions. A standard curve was generated using phosphate concentrations ranging from 0 μM to 50 μM, representing the hydrolysate of ATP. All the phosphate concentration measurements were performed in triplicate. The reaction was performed by incubating the purified UL5–UL52–UL8 complex (250 nM) with ATP (1 mM) in reaction buffer at room temperature for 30 min, in either the presence or absence of 10 μM ssDNA (5′-TTTTTAGCTGGTCATTTTTTTTTTTTTTTTTTTTTTTTTTTTTT-3′). Subsequently, 200 μL of malachite green reagent was added, followed by an additional 30-min incubation at room temperature. The amount of phosphate released from ATP hydrolysis was determined by measuring the optical density at 620 nm using a plate reader (Molecular Devices, SpectraMax iD3). The ATP hydrolysis rates of the UL5–UL52–UL8 complex were then calculated based on the phosphate standard curve. Data were analyzed statistically with GraphPad Prism 9.0.

### Helicase assays

All concentrations listed are final after mixing. Helicase assays were performed using a duplex DNA substrate, which was prepared by heat denaturing and annealing a 20-mer substrate with a 12-bp 5′ tail (5′-TACAGCTACCTAGTCGATGTGCATACTACGGC-3′) and a shorter oligonucleotide labeled with 6-FAM at the 5′ end (FAM-5′-GCCGTAGTATGCACATCGAC-3′) in a 1:1 molar ratio. The substrate (2.5 μM) and protein were incubated in buffer (20 mM HEPES, pH 7.5, 100 mM NaCl, 0.1 mg/mL BSA, 0.1 mM TCEP) at room temperature for 5 min. Premixed assay buffer containing ATP (5 mM), Mg^2+^ (10 mM), and trap DNA (5′-GCCGTAGTATGCACATCGAC-3′, 25 μM) was then added to initiate the reaction. After incubation at 37 °C for a specific time, the reaction was stopped by the addition of one-fifth volume of quench buffer (250 mM EDTA, 40% glycerol, 0.1% bromophenol blue, and 0.1% xylene cyanol). Products were separated by electrophoresis on a 20% non-denaturing acrylamide gel and visualized using a PhosphorImager. The free DNA unwound by the helicase was quantified using ImageJ (v2.9.0) software. All experiments were performed in triplicate, and the data were analyzed using GraphPad Prism 9.0.

### Cryo-EM sample preparation and data collection

Freshly eluted apo UL5–UL52–UL8 from a gel-filtration column was concentrated to ~1.5 mg/mL and mixed with 2 mM ATP, 2 mM MgCl_2_ and 5 μM of a 44-base ssDNA (5′-TTTTTAGCTGGTCATTTTTTTTTTTTTTTTTTTTTTTTTTTTTT-3′). The mixture was incubated on ice for 2 h, and then 3 μL of the solution was applied to cryo-EM AU grids (Quantifoil R1.2/1.3, 300 mesh) that had been glow-discharged at 15 mA for 50 s (0.39 mbar, in air, PELCO easiGlow). The grids were vitrified using a Vitrobot Mark IV (Thermo Fisher Scientific), set to 8 °C and 100% humidity, with a blot force of 3 and a blot time of 5 s. Blotted grids were rapidly plunged into liquid ethane, then transferred to liquid nitrogen and stored at cryogenic temperatures prior to screening and data collection.

Cryo-EM data were collected on a Titan Krios G4 microscope operating at 300 kV, equipped with a Gatan K3 direct electron detector and a GIF BioQuantum energy filter (Core Facility of Wuhan University). Images were acquired using Thermo Scientific EPU software in counting mode at a nominal magnification of 105,000×, with 40 frames per movie, an accumulated dose of 50 electrons per Å², and a pixel size of 0.84 Å. The defocus was set between –0.8 μm and –2.5 μm. A total of 8249 movies were collected.

### Cryo-EM image processing

All datasets were processed within CryoSPARC. The images were gain-corrected and dose-weighted. Motion correction and contrast transfer function (CTF) estimation were performed using the patch-based motion correction and patch CTF estimation programs in CryoSPARC Live^55^. Initial particle picking was conducted using the blob picker and inspect picker functions in CryoSPARC, yielding ~9,018,206 particles within a diameter range of 70–200 Å. These particles were then subjected to 2D classification. After three rounds of 2D classification, 1,065,699 particles representing diverse views were selected for Ab initio reconstruction followed by Heterogeneous Refinement. This refinement separated out 433,495 particles, which underwent Non-uniform Refinement, resulting in a global map with poorly resolved density in the helicase and part of the primase regions. An additional round of Heterogeneous Refinement and Non-uniform Refinement was performed, generating a new map that showed significantly improved density for the helicase and primase. We further performed 3D classification, and the best class was selected for Non-uniform Refinement. This was subsequently refined using local CTF refinement. The Non-uniform Refinement yielded a global resolution of 3.47 Å, as determined by the gold-standard Fourier shell correlation (FSC = 0.143) criterion; this map is referred to as the HPF. To further improve map quality, local refinement was performed for the UL5–UL52_NTD-hinge_ domain using a mask generated in ChimeraX^61^, resulting in a locally refined map at 3.34 Å resolution, designated HP.

### Model building and refinement

Initial models for UL5, UL52, and UL8 were generated using AlphaFold2^62^. UL8, UL52_AEP_, UL52_NTD-hinge_, and individual domains of UL5 were rigidly docked into the density map using ChimeraX. The obtained model was manually adjusted in COOT^63^, where ADP, Mg^2+^ ion and the DNA strand were built. The structure was subjected to real-space refinement in PHENIX^64^ with Ramachandran and secondary structure restraints applied. The model was iteratively corrected for local fit in COOT and refined over multiple rounds. Structural figures were prepared using PyMOL (www.pymol.org/) and ChimeraX. Secondary structure elements were mapped onto the sequence through ESPript3.0.

## Supplementary information

Supplementary information

Supplementary Video S1

## Data Availability

All data needed to evaluate the conclusions are present in the paper and/or Supplementary information. The atomic coordinate and cryo-EM density map of HSV-1 helicase-primase are deposited in the Protein Data Bank with accession codes 9VLQ and EMD-65163 (composite map), EMDB-66328 (HPF Consensus map), EMDB-66330 (HP focused map).
