# Supplementary material for: Structural and mechanistic insights into the herpes simplex virus type 1 helicase-primase primosome
Source: Cell Discov. 2025 Dec 10;11:100. doi: 10.1038/s41421-025-00855-4 (PMC12695949; doi:10.1038/s41421-025-00855-4)
Supplement: Supplementary file 1 — Supplementary information [file 41421_2025_855_MOESM1_ESM.pdf]

Supplementary information for

Structural and mechanistic insights into the herpes simplex virus type 1 helicase-primase primosome

Yaqi Wu[1,2], Ziyi Jiang[1,2], Xiaoling Chen[1,2], Danyang Li[3], Zhengyu Zhang[1,2]*, Changjiang Dong[1,2]*

1. Department of Thyroid and Breast Surgery, Zhongnan Hospital of Wuhan University, State Key Laboratory of Virology, School of Pharmaceutical Sciences, Wuhan University, Wuhan 430071, China
2. Key Laboratory of Combinatorial Biosynthesis and Drug Discovery, Ministry of Education, School of Pharmaceutical Sciences, Wuhan University, Wuhan 430071, China
3. The Cryo-EM Center, Core facility of Wuhan University, Wuhan University, Wuhan 430071, China

*Corresponding author

Email: zhengyu.zhang@whu.edu.cn; changjiangdong@whu.edu.cn

The file includes:

Supplementary Figures S1 to S11

Supplementary Video 1

Supplementary Table S1

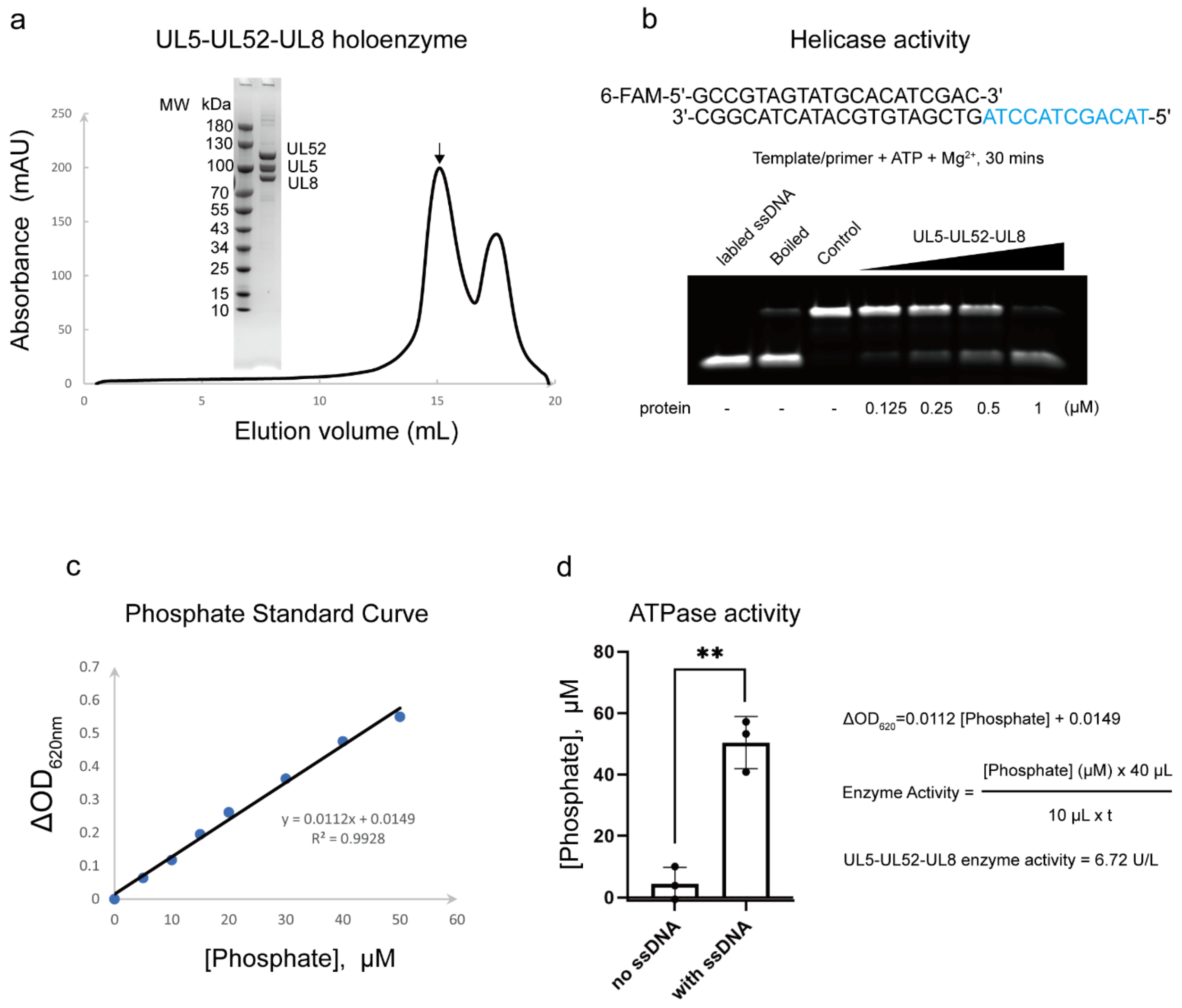

Supplementary Fig. S1 | Biochemical characterization of the HSV-1 UL5-UL52-UL8 primosome.

a Protein purification was analyzed by size-exclusion chromatography (SEC) and sodium dodecyl sulfate–polyacrylamide gel electrophoresis (SDS-PAGE). **b** Representative results of the in vitro helicase assay. The assay, conducted with ATP and Mg^{2+} for 30 minutes, shows that the protein exhibits unwinding activity across a range of concentrations. **c-d** ATPase activity of the purified protein measured using a commercial enzymatic assay kit according to the manufacturer's protocol.

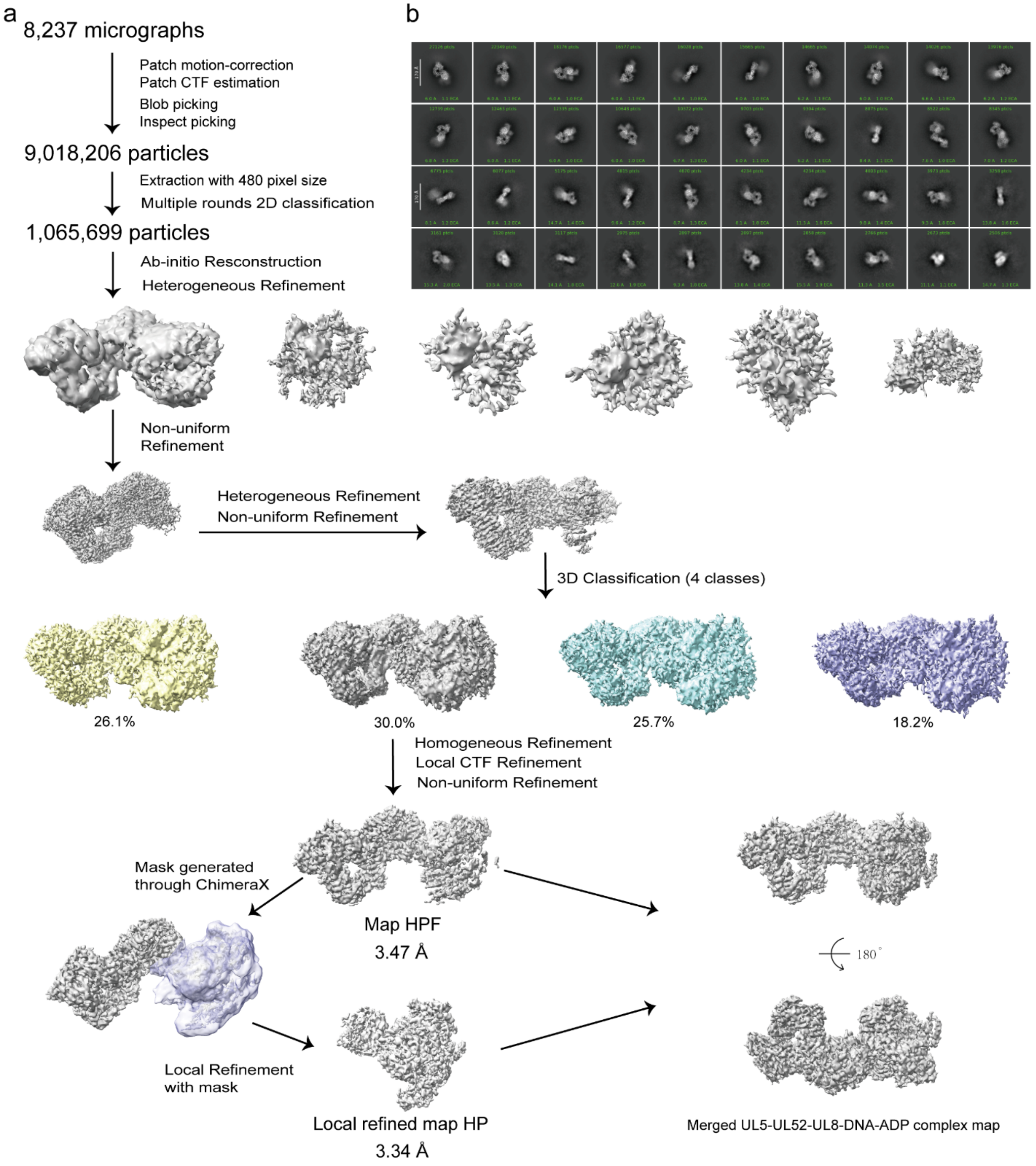

Supplementary Fig. S2 | Flowchart of cryo-EM single particle data processing and structure determination of the HSV-1 UL5-UL52-UL8 complex. a Data processing pipeline. **b** Representative 2D classes of the UL5-UL52-UL8 complex.

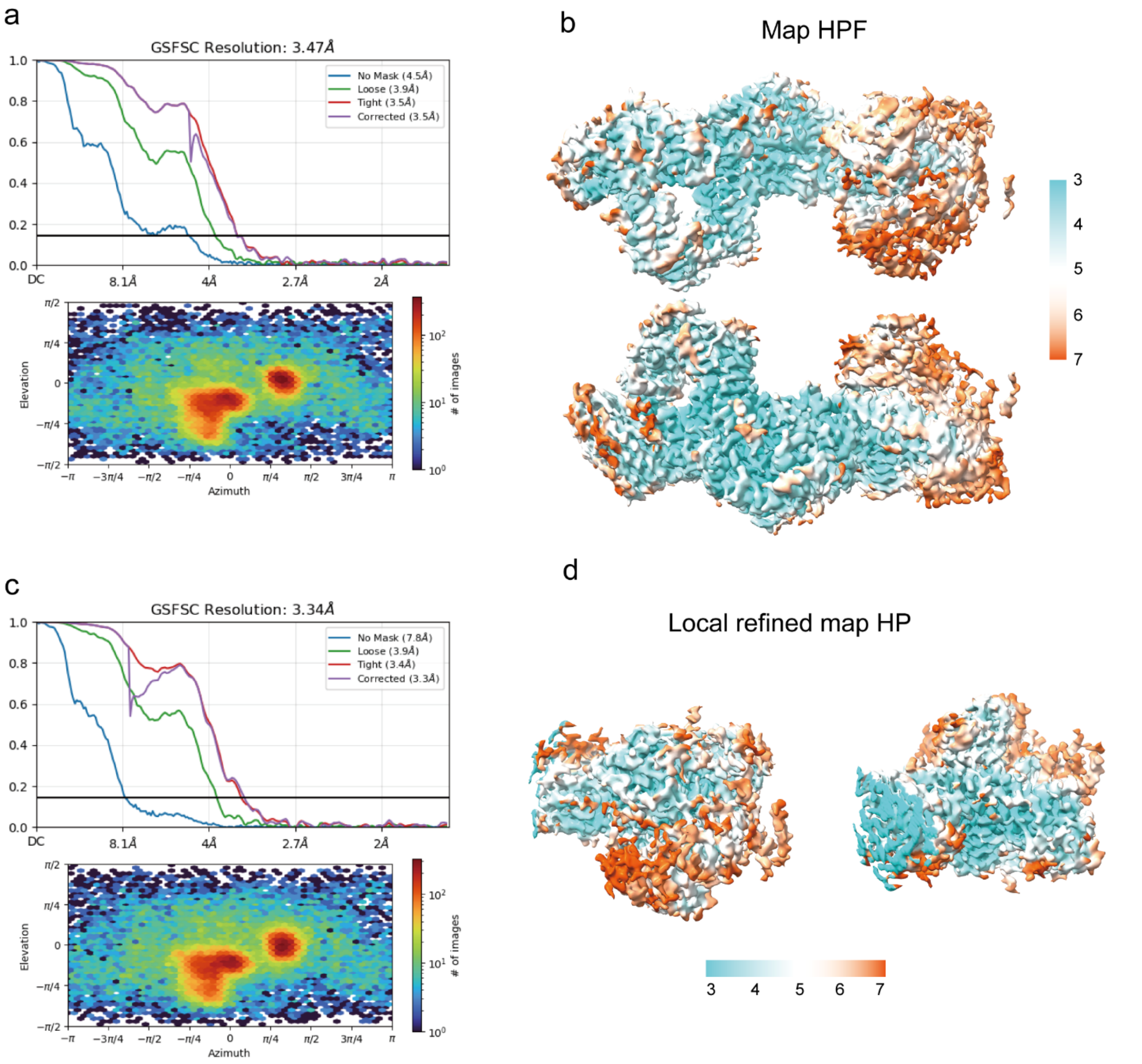

Supplementary Fig. S3 | Local resolution analysis and model validation of the HSV-1 UL5-UL52-UL8 complex. a Gold-standard Fourier shell correlation (FSC) curve and angular distribution of particle projections for the HPF reconstruction. **b** Local resolution map of the HPF reconstruction at a volume threshold of 0.1. **c** Gold-standard FSC curve and angular distribution of particle projections for the locally refined HP subregion. **d** Local resolution map of the locally refined HP subregion at a volume threshold of 0.1.

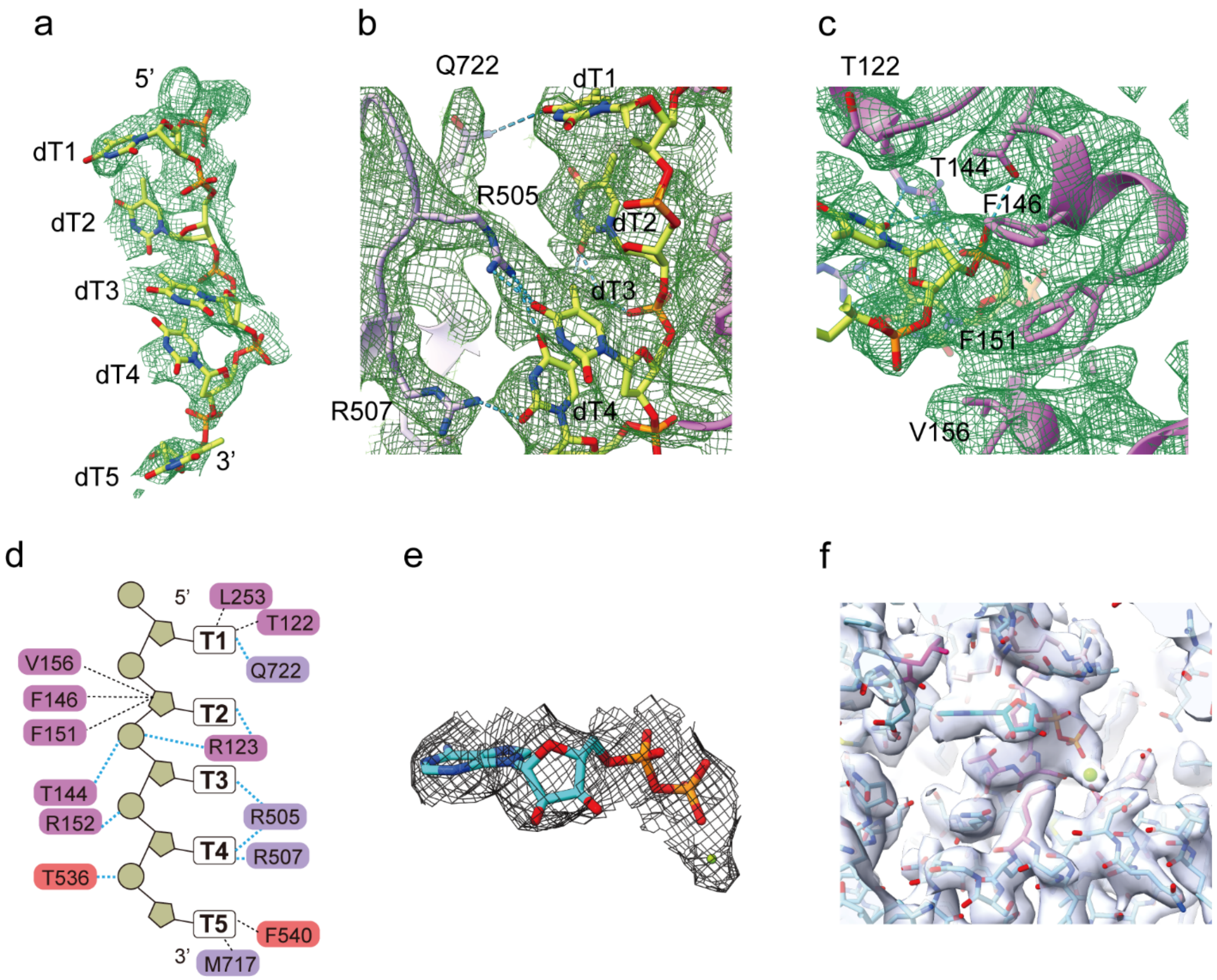

Supplementary Fig. S4 | Representative cryo-EM densities for key functional sites. a Density for the bound ssDNA. **b-c** Representative close-up views of the map for key amino acid residues interacting with the ssDNA. **d** Schematic representation of the UL5-ssDNA interaction. Blue dashed lines represent polar interactions, and black dashed lines indicate hydrophobic interactions. **e** Density for the bound ADP molecule and the coordinating Mg^{2+} ion. **f** Close-up views of the map for key amino acid residues around the ADP and Mg^{2+}.

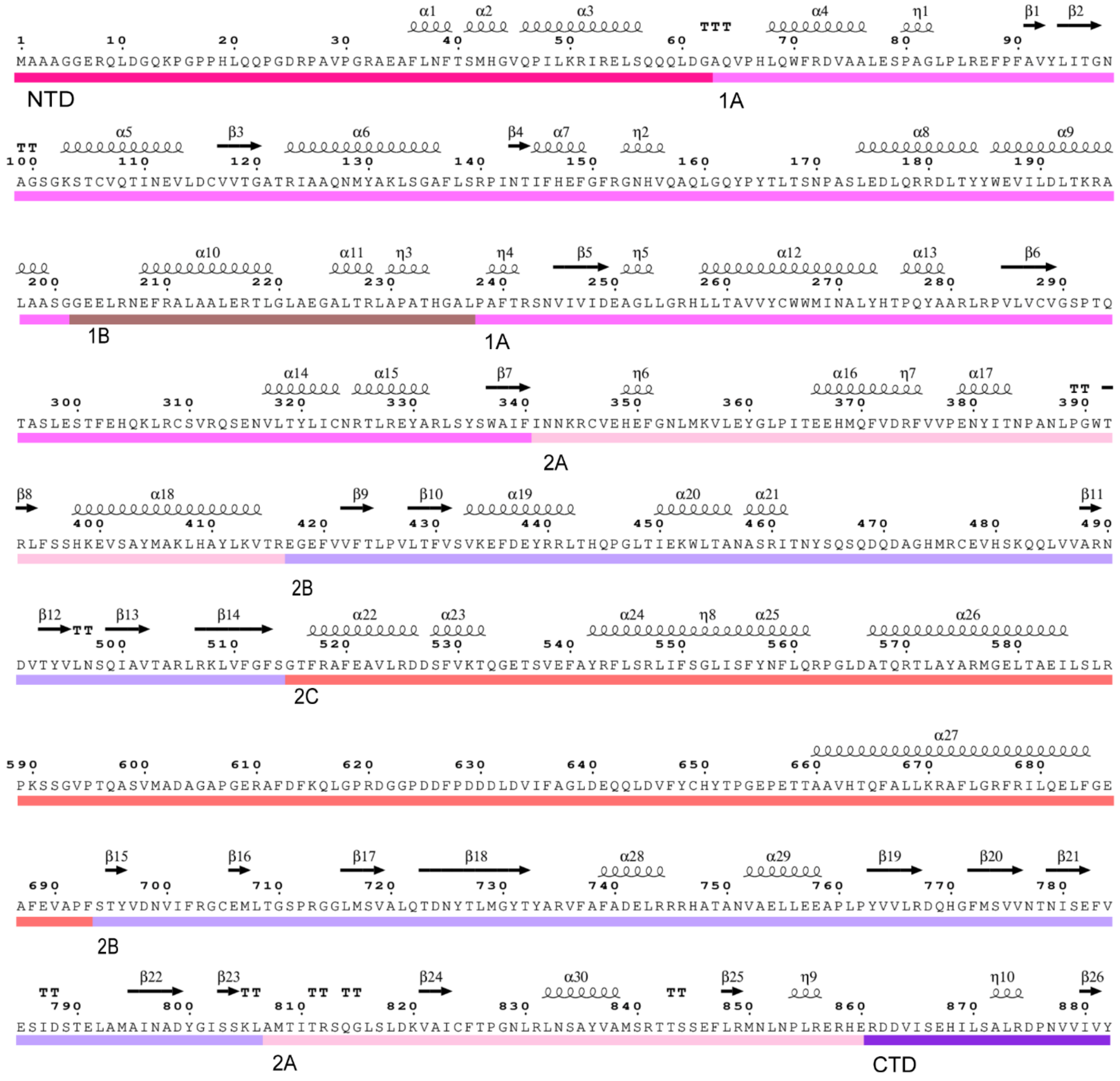

Supplementary Fig. S5 | Domain architecture and secondary structure mapping of HSV-1 helicase subunit UL5. Amino acid sequence annotated with domain-specific coloring. Secondary structure elements: α-helices (cartoons) and β-strands (arrows) mapped onto the sequence. Residue numbering corresponding to secondary structure assignments.

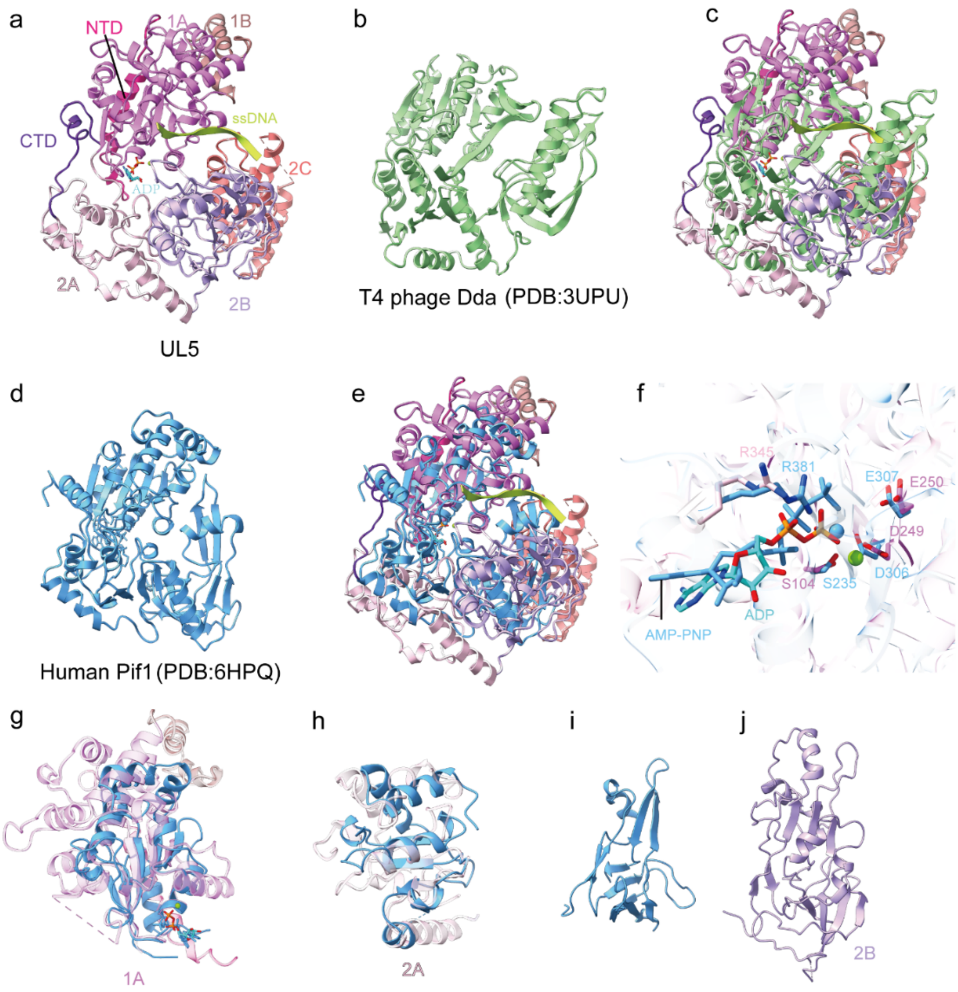

Supplementary Fig. S6 | Structural architecture of UL5 helicase and comparison with SF1B family homologues. a Cartoon representation of UL5 bound to ADP and ssDNA colored according to the domain schematic. **b** Cartoon representation of the crystal structure of the SF1B family helicase Dda from the T4 phage (3UPU). **c** Structural superposition of Dda with UL5. **d** Cartoon representation of the crystal structure of the SF1B family helicase human Pif1 in complex with AMP-PNP (6HPQ). **e** Structural superposition of human Pif1 with UL5. **f** Conserved ATPase active site revealed by Pif1-UL5 alignment. **g** Structural alignment of domain 1A between UL5 and Pif1. **h** Structural alignment of domain 2A between UL5 and Pif1. **i** 2B domain of Pif1. **j** 2B domain of UL5.

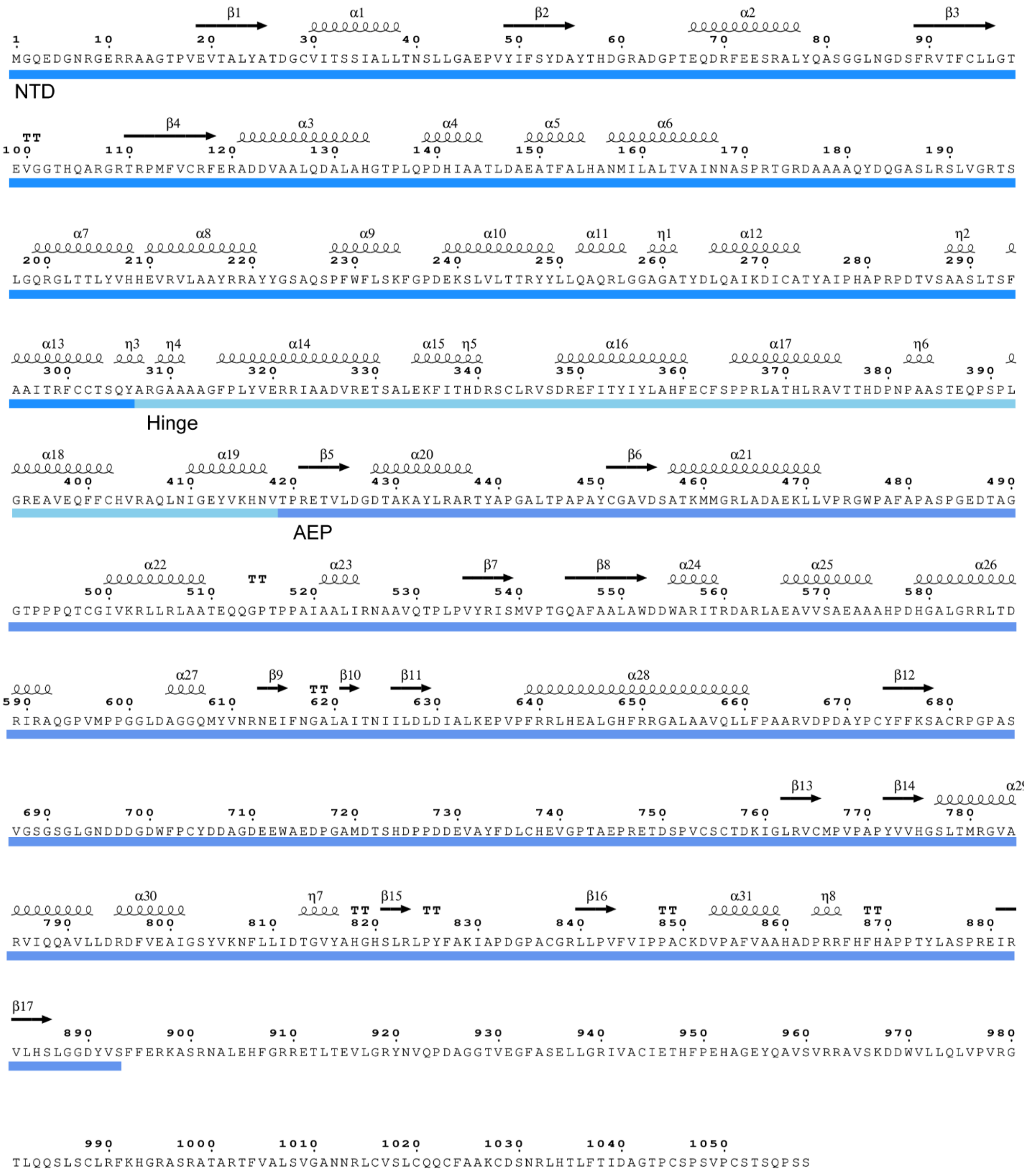

Supplementary Fig. S7 | Domain architecture and secondary structure mapping of the HSV-1 primase subunit UL5. Amino acid sequence annotated with domain-specific coloring. Secondary structure elements: α-helices (cartoons) and β-strands (arrows) mapped onto the sequence. Residue numbering corresponding to secondary structure assignments.

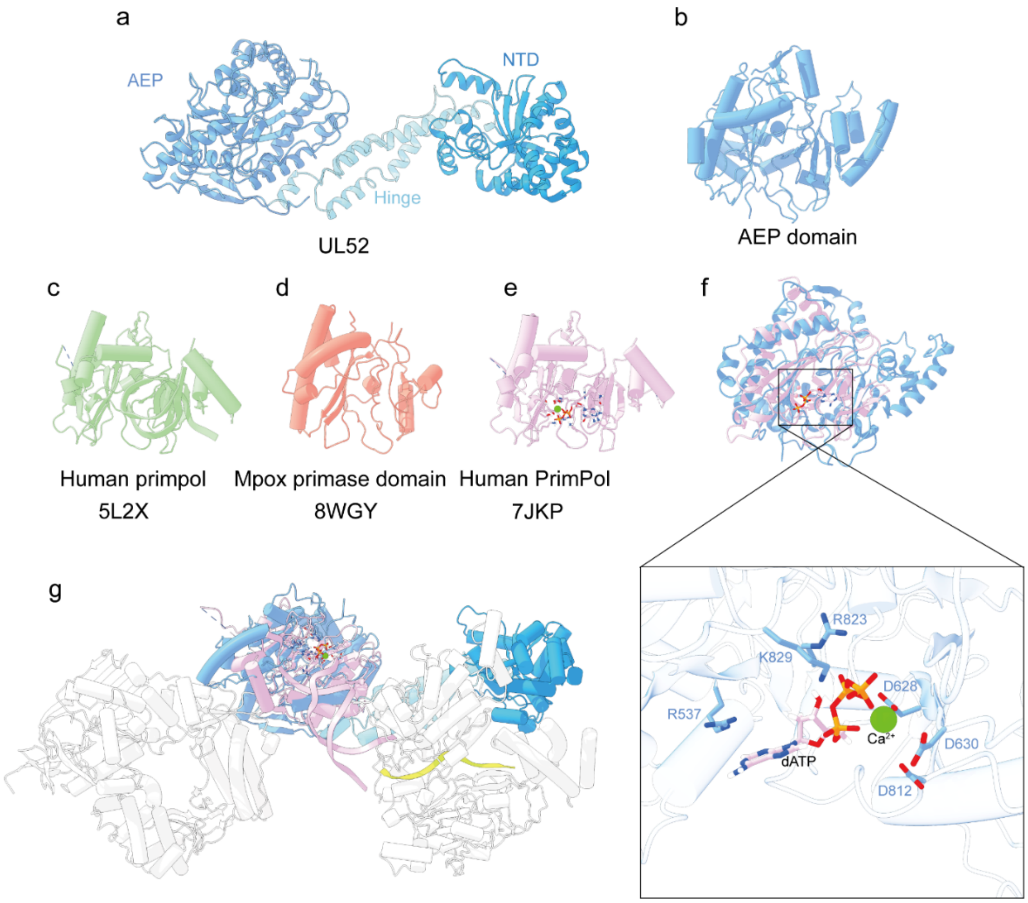

Supplementary Fig. S8 | Structural architecture of the HSV-1 primase subunit UL52 and comparative analysis. a Domain organization of UL52 (cartoon representation; schematic coloring). The C-terminal region (residues 894–1058) shows poorly resolved density. **b** Cartoon representation of the AEP domain of UL52. **c** Cartoon representation of the crystal structure of the human PrimPol ternary complex. **d** Cartoon representation of the primase domain of mpox virus. **e** Structure of human PrimPol complexed with dATP and Ca^{2+} (PDB: 7JKP), dsDNA omitted for clarity. **f** Structural superposition of the UL52 AEP domain and human PrimPol (bound to dATP and Ca^{2+}). dATP occupies a pocket formed by UL52 residues Lys829, Arg823, Arg537, Asp630, Asp628, and Asp812. **g** Structural superposition of the UL5-UL52-UL8 primosome complex structure and 7JKP with dsDNA shown as cartoon.

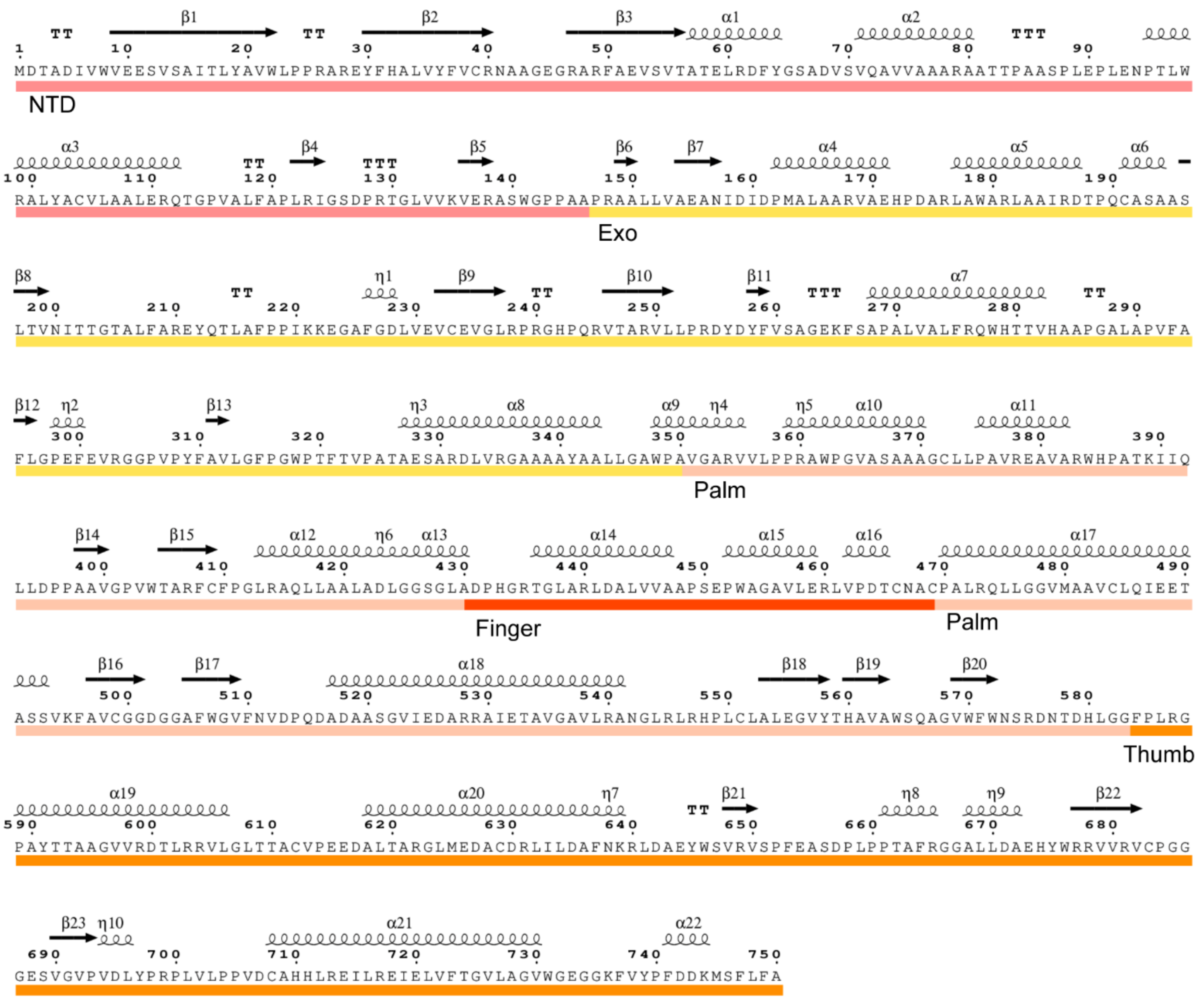

Supplementary Fig. S9 | Domain architecture and secondary structure mapping of the HSV-1 accessory protein subunit UL8. Amino acid sequence annotated with domain-specific coloring. Secondary structure elements: α-helices (cartoons) and β-strands (arrows) mapped onto the sequence. Residue numbering corresponding to secondary structure assignments.

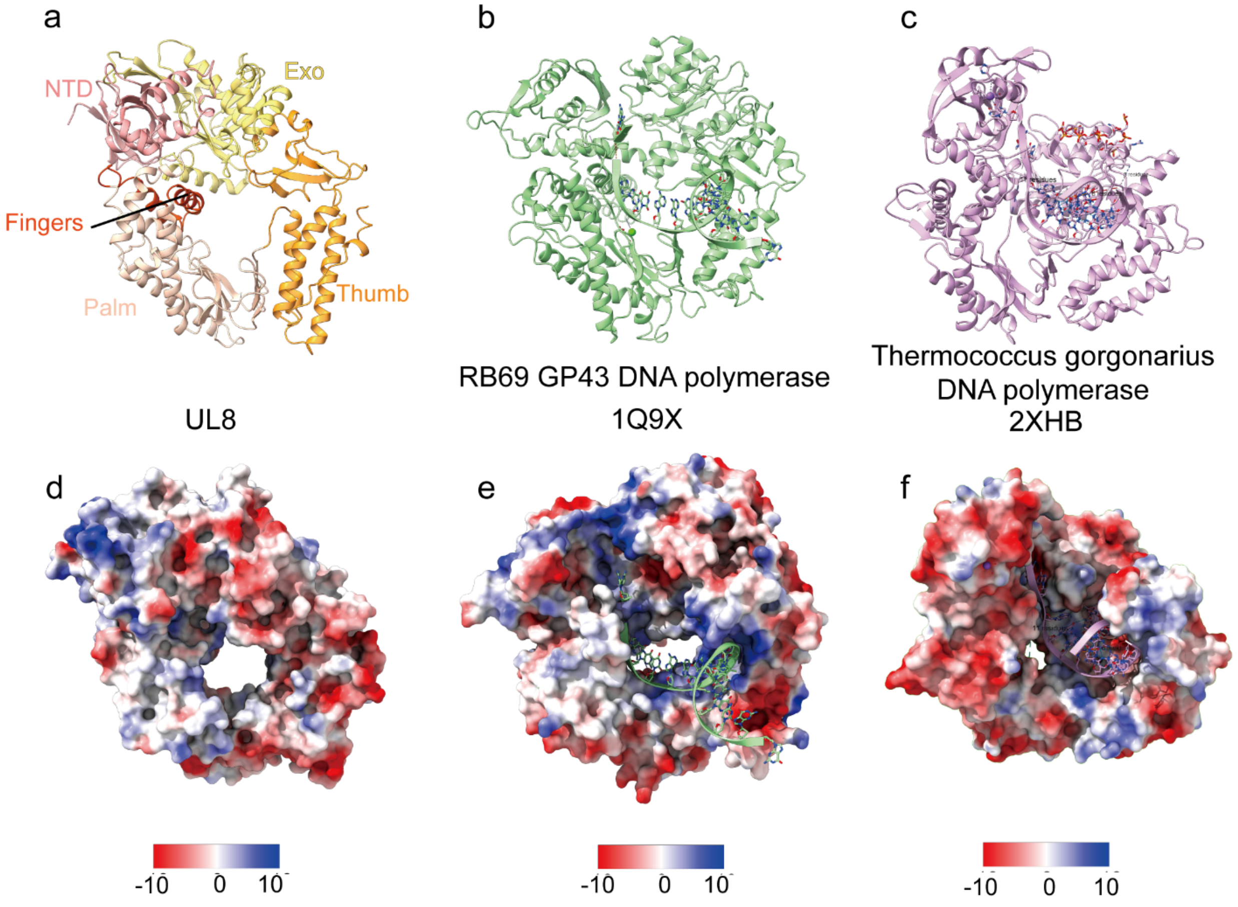

Supplementary Fig. S10 | Overall structure of the HSV-1 accessory protein subunit UL8. a Cartoon representation of UL8 colored by domain (schematic). **b** Structure of the *Enterobacteri* phage RB69 GP43 DNA polymerase (PDB code 1Q9X). **c** Structure of *Thermococcus gorgonarius* DNA polymerase (PDB code 2XHB). (blue: electropositive; red: electronegative). **d** Charge distribution on the surfaces of UL8. **e** Charge distribution on the surfaces of the *Enterobacteri* phage RB69 GP43 DNA polymerase. (blue: electropositive; red: electronegative). **f** Charge distribution on the surfaces of *Thermococcus gorgonarius* DNA polymerase. (blue: electropositive; red: electronegative).

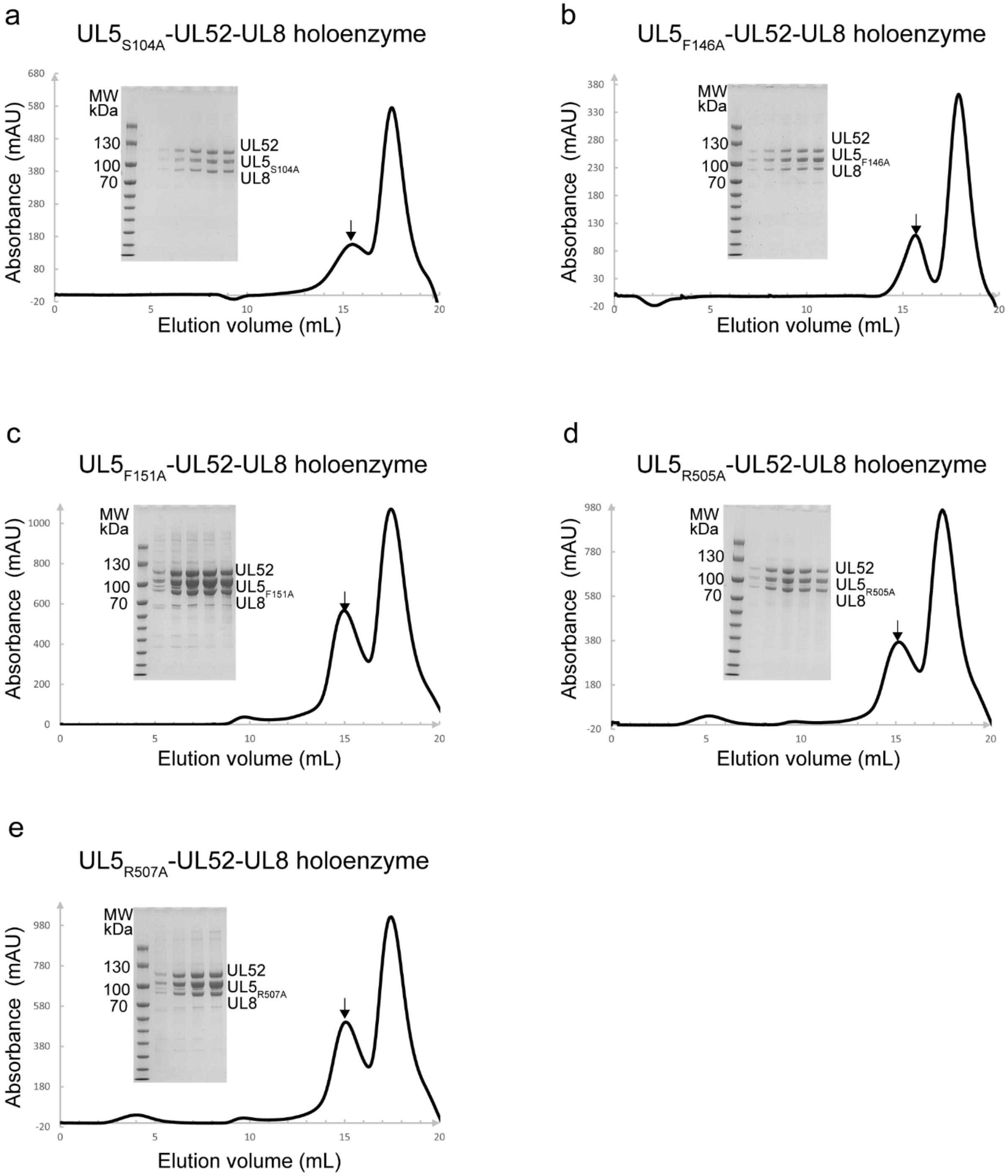

Supplementary Fig. S11 | Purification and characterization of the mutant proteins. Size-exclusion chromatography profiles and SDS-PAGE analysis for mutants (a) Ser104Ala, (b) Phe146Ala, (c) Phe151Ala, (d) Arg505Ala, and (e) Arg507Ala. All proteins were purified to high purity and eluted as monomers under the experimental conditions.

Supplementary Video 1 | 3D visualization of HSV-1 primosome structural flexibility. Main flexible domains are labeled. HSV-1 primosome conformational changes when presented with ATP, Mg^{2+}, and ssDNA. The DNA-binding groove constricts when the UL5 1A and 2A–2B domains rotate clockwise, and expands when they rotate counterclockwise. The primase NTD moves concordantly with the helicase domains, whereas the accessory subunit shifts slightly in the opposite direction. Overall, the helicase subunits, together with the primase NTD and hinge domain, exhibit substantially higher mobility than the primase AEP domain and accessory subunits.

Supplementary Table S1 | Data processing and refinement

	UL5-UL52-UL8 Composite map (PDB 9VLQ) (EMDB-65163)	UL5-UL52-UL8 HPF Consensus map (EMDB-66328)	UL5-UL52-UL8 HP focused map (EMDB-66330)
**Data collection**			
Microscope	Krios G4	Krios G4	Krios G4
Detector	Gatan K3	Gatan K3	Gatan K3
Voltage (kV)	300	300	300
Defocus range (μm)	-0.8 to-2.5	-0.8 to-2.5	-0.8 to-2.5
Pixel size (Å)	0.84	0.84	0.84
Electron exposure (e-/ Å^2)	50	50	50
Particles (initial)	1065699	1065699	1065699
Particles (final)	35958	35958	35958
**Reconstruction**			
Symmetry		C1	C1
Map Resolution (unmasked; Å)		4.5	7.8
Map Resolution (masked; Å)		3.47	3.34
FSC threshold		0.143	0.143
**Model composition**			
Nonhydrogen atoms	17678		
Protein residues	2275		
DNA nucleotides	5		
Ions	1 Mg^{2+}		
Ligands	1 ADP		
**Refinement**			
B factor (Å^2)	133.64/106.50/98.83 (Protein/DNA/ADP)		
R.ms. deviations			
Bonds (Å)	0.002		
Angles (°)	0.471		
**Validation**			
MolProbity score	1.82		
Clashscore	7.59		
Rotamer outliers (%)	0.00		
Ramachandran plot (% favored)	94.02		
Ramachandran plot (% outliers)	0.00		
